# An Unusual Presentation of a Completely Transected Aorta Presenting As Acute Limb Ischemia With Intramural Thrombus

**DOI:** 10.7759/cureus.30754

**Published:** 2022-10-27

**Authors:** Swarnava Tarafdar, Raja Lahiri, Ajay Kumar, Aakanksha Agarwal, Garima Sharma

**Affiliations:** 1 Radiodiagnosis & Imaging, All India Institute of Medical Sciences, Rishikesh, Rishikesh, IND; 2 Cardiothoracic & Vascular Surgery, All India Institute of Medical Sciences, Rishikesh, Rishikesh, IND; 3 Trauma Surgery & Critical Care, All India Institute of Medical Sciences, Rishikesh, Rishikesh, IND

**Keywords:** angiography, multidetector computed tomography, intraluminal thrombus, inferior vena cava injury, traumatic aortic injury

## Abstract

Contrast-enhanced multidetector computed tomography is currently the preferred imaging modality to diagnose acute traumatic aortic injury (ATAI). Rarely, ATAI can present with atypical findings that make the diagnosis and further management exceptionally challenging. Furthermore, ATAI can also be associated with inferior vena cava injury showing only indirect signs on later imaging. We present an unusual case of traumatic aortic disruption mimicking acute limb ischemia.

## Introduction

Acute traumatic aortic injury (ATAI) is a severe condition with high pre-hospital mortality. The mortality rate is up to 95% and 30% if treated early [1,2]. The fast and readily available multidetector computed tomography (MDCT) is the preferred imaging modality to diagnose this serious condition [3]. The isotropic data acquired with MDCT can generate multiplanar reformations that are extremely useful for surgical planning [4]. MDCT has a high negative predictive value of up to 100%; therefore, it is considered the sole imaging technique to exclude traumatic aortic rupture [1]. Conventional angiography is reserved for cases with equivocal findings on CT; however, its role alone is limited [4]. Non-penetrating aortic injury is a relatively uncommon diagnosis presenting in an emergency setting. However, it is a frequent cause of death in motor vehicle accidents, with an incidence of up to 20% of road traffic accident-related deaths [5].

## Case presentation

**Figure 1:**
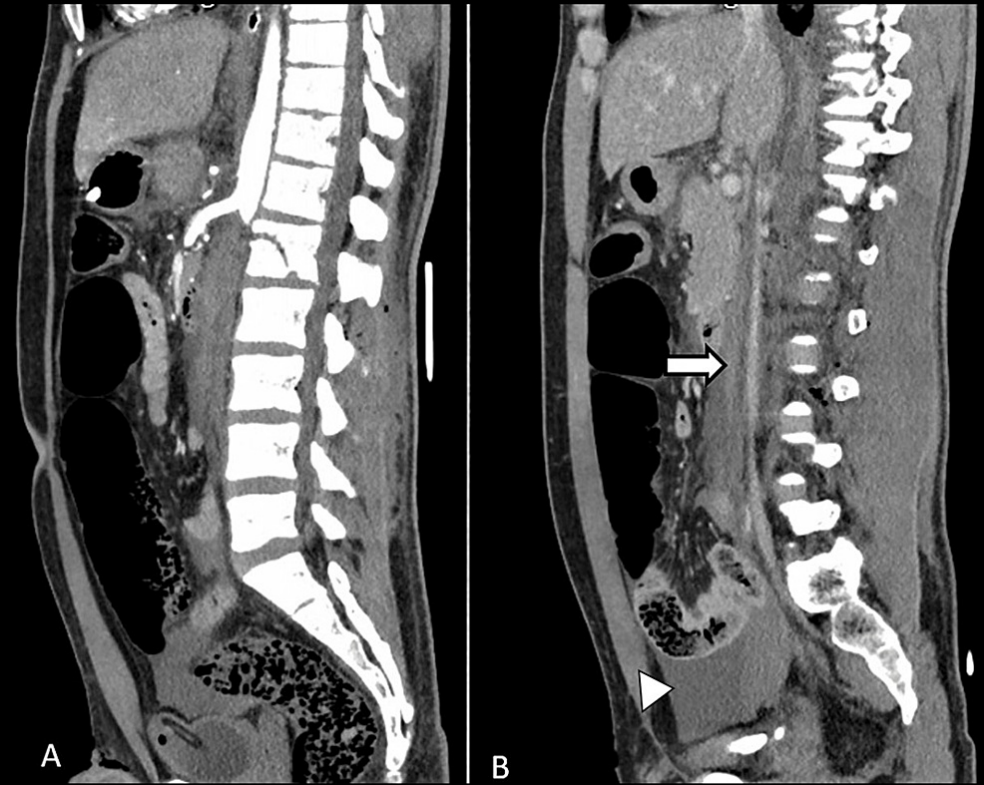
A. Complete occlusion of abdominal aorta distal to the origin of the inferior mesenteric and right renal artery with intraluminal thrombus and associated fracture of L1 vertebra. B. Inferior vena cava (arrow) is narrow in caliber showing post-contrast homogenous opacification with no apparent defect or active contrast extravasation. Mild free intraperitoneal hematoma is seen in the pelvis (arrowhead).

**Figure 2:**
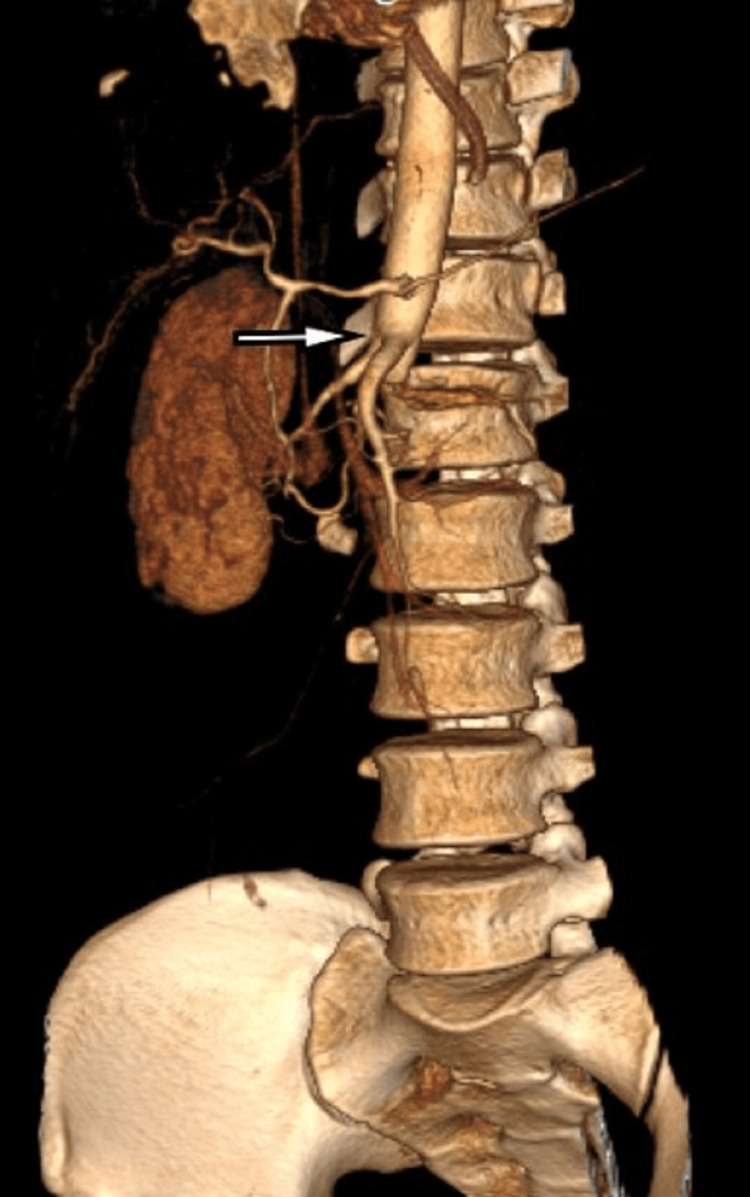
Volume rendering technique image showing occlusion of the aorta distal to the origin of the right renal artery (arrow). The left renal artery was also occluded.

**Figure 3:**
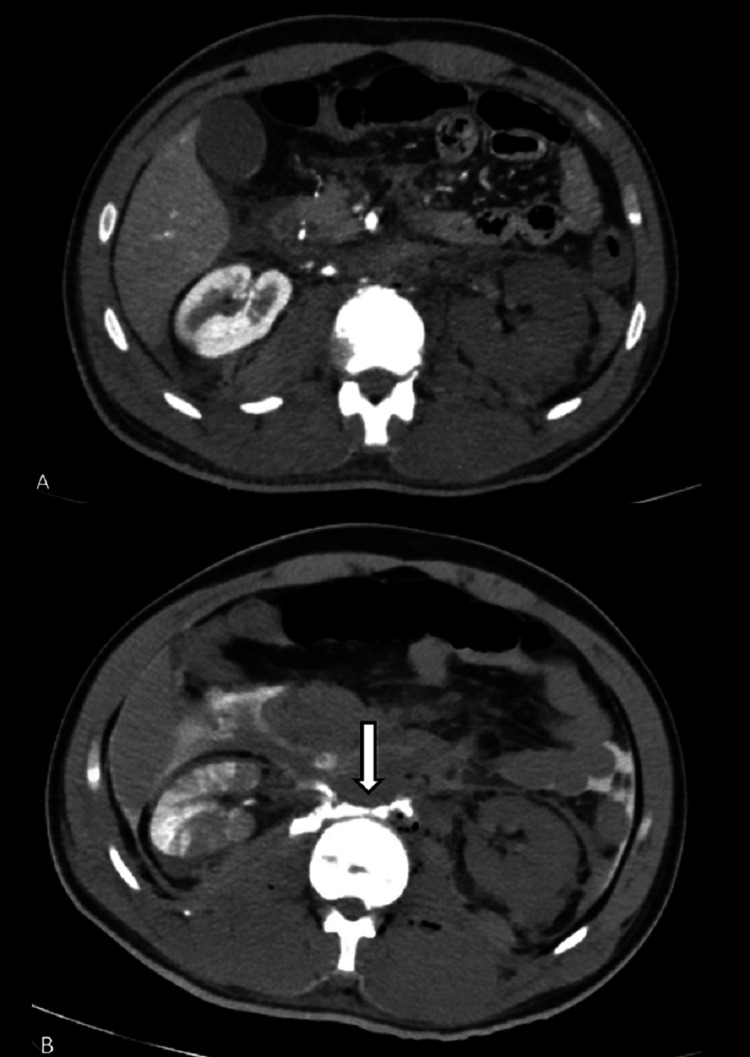
A. No significant periaortic hematoma seen on the initial multidetector computed tomography at the site of aortic occlusion. The left kidney is not showing contrast opacification due to occlusion of left renal vessels. B. On re-imaging with plain computed tomography, the contrast was selectively delivered through an embolectomy catheter placed at the upper extent of the thrombus and showed extravasation of contrast in intraperitoneal and retroperitoneal space from the aorta (arrow).

**Figure 4:**
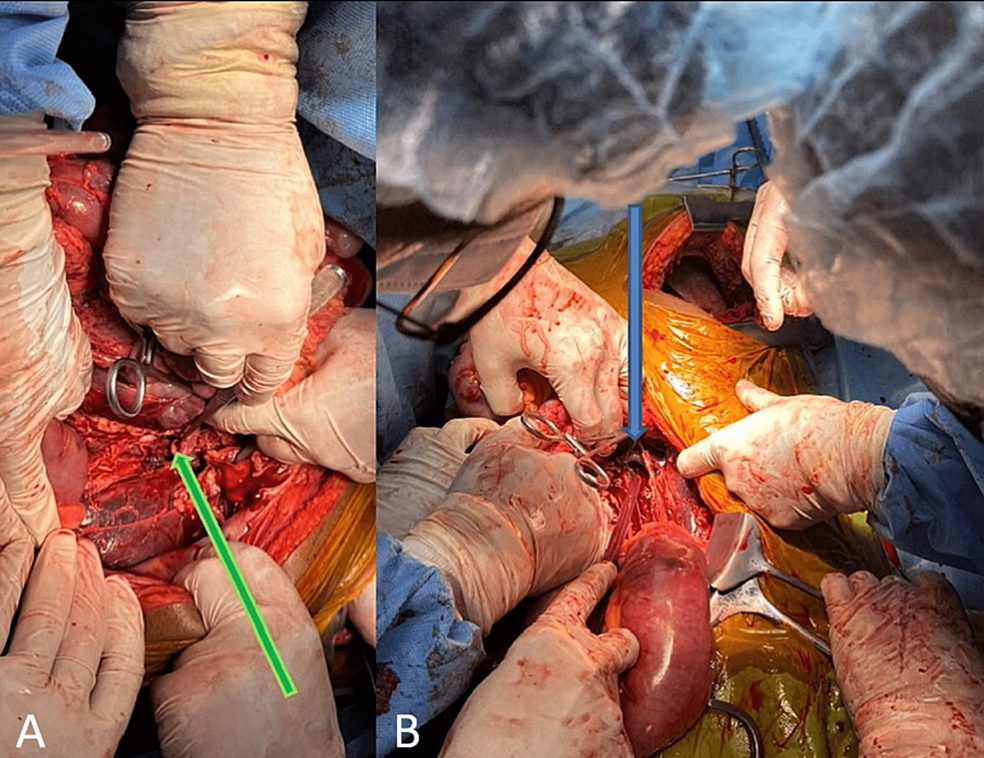
A. Intraoperatively, the tip of forceps shows a proximal transected aorta (arrow). B. Left renal vein visible with a clamp in proximal inferior vena cava (arrow).

A 26-year-old male patient presented in Trauma & Emergency with a history of a motor vehicle accident. On presentation, the patient was hemodynamically stable, and the focused assessment of sonography in trauma (FAST scan) was negative. X-ray showed a non-displaced L1 vertebral body fracture. On clinical evaluation, both lower limb pulses were absent with markedly reduced temperature. Color Doppler of both lower limbs showed monophasic flow in femoral, popliteal, anterior tibial, posterior tibial, and dorsalis pedis arteries. The patient was immediately referred for MDCT on a Philips 64-slice scanner which showed non-opacification of the entire infrarenal aorta after the origin of the right renal artery (Figures 1A, 2). The left renal artery and vein were also occluded, leading to a completely devascularized left kidney (Figure 3A). Aortic continuity appeared to be maintained, and there was no significant periaortic hematoma. The inferior vena cava (IVC) was narrow in caliber throughout its entire length (Figure 1B). In view of clinical and radiological findings, the patient was taken for an emergency embolectomy. A large amount of thrombus was recovered from bilateral iliac arteries and abdominal aorta. However, post-embolectomy, blood flow remained sluggish. This led to clinical suspicion of a possible intimal tear, and a re-imaging was done with contrast delivered selectively through the embolectomy catheter placed at the upper extent of the thrombus. The CT scan showed extravasation of contrast in intraperitoneal and retroperitoneal space through a defect in the aortic wall which was considered previously to be sealed by the occluding thrombus within the aorta (Figure 3B). The patient was immediately taken up for an emergency exploratory laparotomy. On exploration, the aorta was found to be completely transected distal to the origin of the right renal artery with a gap of around 6-7 cm between the proximal and distal segments (Figure 4). Another injury was found in IVC at the junction of the left renal vein with a defect of approximately 2.5 × 2 cm in the IVC. There was a significant intraperitoneal collection of blood at the time of exploration. Because the patient expired after the surgery due to blood loss, spinal cord status could not be evaluated by follow-up MRI.

## Discussion

Presentation of such a case to an emergency department is rare. What complicates it further is that such patients present with diverse symptoms. The most common presentation is hemorrhagic shock and other findings such as a positive FAST scan or an expanding retroperitoneal hematoma. In this case, color Doppler showed monophasic flow in lower limb arteries prompting immediate MDCT to rule out vascular occlusion. These are often associated with a significant injury to other vital organs such as the head, chest, and other solid organ injuries as these often involve a high-energy transfer. Blunt aortic injury can be categorized based on the severity of the aortic injury. Type I aortic injury is an intimal tear, Type II injury is an intramural hematoma, Type III is a pseudoaneurysm, and Type IV is an aortic rupture [6].

The direct signs while using MDCT for ATAI may include pseudoaneurysm formation, vessel wall disruption, intimal flap, intramural hematoma or thrombus, and active extravasation of contrast. Furthermore, the indirect imaging signs on imaging are periaortic hematoma, change in aortic caliber, and irregular aortic contour [3]. More severe injuries can have both direct and indirect signs [3]. Injury of the isthmus portion of the aorta is more common than the involvement of descending aorta in ATAI [2,4]. Injuries can be associated with diaphragmatic injury and fracture of the thoracic vertebra. In cases of traumatic IVC injuries, the signs on imaging include contour abnormality or intimal flap, IVC thrombosis or intracaval fat, and retroperitoneal hematoma [7].

The diagnostic accuracy of MDCT in ATAI is up to 100% in various studies, sensitivity is more than 98%, and specificity is up to 100% [2]. Some studies have argued that MDCT may be better than catheter angiography [8,9]. Traumatic rupture involving descending abdominal aorta associated with IVC injuries is rare [1,8,9]. IVC injury is usually associated with a hepatic laceration in up to 80% of cases [7]. However, in this case, no association of hepatic or other visceral organs was noted. If the patient’s proximal lumen of the aorta shows a flame-shaped appearance, possible traumatic dissection followed by thrombosis can be considered and discussed in the differential diagnosis. In addition, none of the direct signs of IVC injury were seen in our case. The intramural thrombus is a less common direct sign of aortic injury. Therefore, pseudoaneurysm formation or active contrast extravasation may be absent even in cases with significant traumatic aortic defect due to the sealing of the gap by intramural thrombus. Moreover, periaortic hematoma being an indirect sign has previously resulted in high false-positive results [1]. These clinical and radiological findings can lead to diagnostic errors and can affect patient management and outcomes. Direct injury of the aorta may also result from penetrating injury of vertebral body fractures, as was likely in our case [4]. There was a fracture of the L1 vertebral body in our case, which probably resulted in an aortic rupture. This probably led to a large aortic tear followed by the formation of an intraluminal thrombus which led to the temporally sealing of the tear by thrombus obscuring the direct signs of trauma. The tear was reopened following the thrombectomy leading to a massive bleed. Therefore, in the case of vertebral fractures with aortic thrombus, the possibility of aortic rupture should be ruled out and large aortic rupture may be sealed off due to thrombus.

## Conclusions

Although fatal, some cases of ATAI do reach the hospital on time. Diagnosis in most cases is straightforward, and the need for emergency surgical exploration is evident. This case showed us how ATAI in rare instances can present differently, making the diagnosis difficult and management challenging. The possibility of aortic injury should be kept in mind in trauma victims with spinal injuries with the rare absence of definite signs, as in this case, and keeping high suspicion will prevent diagnostic errors.

## References

[1] Steenburg SD, Ravenel JG (2008). Acute traumatic thoracic aortic injuries: experience with 64-MDCT. AJR Am J Roentgenol.

[2] Leon M, Chavez LO, Chavez A, Surani S (2020). Blunt aortic / inferior vena cava injury: are we consistently providing the same level of care?. Cureus.

[3] Cullen EL, Lantz EJ, Johnson CM, Young PM (2014). Traumatic aortic injury: CT findings, mimics, and therapeutic options. Cardiovasc Diagn Ther.

[4] Steenburg SD, Ravenel JG, Ikonomidis JS, Schönholz C, Reeves S (2008). Acute traumatic aortic injury: imaging evaluation and management. Radiology.

[5] Schulman CI, Carvajal D, Lopez PP, Soffer D, Habib F, Augenstein J (2007). Incidence and crash mechanisms of aortic injury during the past decade. J Trauma.

[6] Lee WA, Matsumura JS, Mitchell RS (2011). Endovascular repair of traumatic thoracic aortic injury: clinical practice guidelines of the Society for Vascular Surgery. J Vasc Surg.

[7] Tsai R, Raptis C, Schuerer DJ, Mellnick VM (2016). CT appearance of traumatic inferior vena cava injury. AJR Am J Roentgenol.

[8] Mirvis SE, Shanmuganathan K (2007). Diagnosis of blunt traumatic aortic injury 2007: still a nemesis. Eur J Radiol.

[9] Ng CJ, Chen JC, Wang LJ, Chiu TF, Chu PH, Lee WH, Wong YC (2006). Diagnostic value of the helical CT scan for traumatic aortic injury: correlation with mortality and early rupture. J Emerg Med.

